# High-Sensitivity Flexible Piezoresistive Pressure Sensor Using PDMS/MWNTS Nanocomposite Membrane Reinforced with Isopropanol for Pulse Detection

**DOI:** 10.3390/s22134765

**Published:** 2022-06-24

**Authors:** Zhiming Long, Xinggu Liu, Junjie Xu, Yubo Huang, Zhuqing Wang

**Affiliations:** 1 Med + X Center for Manufacturing, West China Hospital of Sichuan University, Chengdu 610041, China; 2021223020054@stu.scu.edu.cn; 2 School of Mechanical Engineering, Sichuan University, Chengdu 610065, China; 2020223020005@stu.scu.edu.cn (X.L.); 2020223025070@stu.scu.edu.cn (J.X.); huangyubo@scu.edu.cn (Y.H.)

**Keywords:** flexible pressure sensor, piezoresistive, PDMS, MWNTS, scanning circuit

## Abstract

Flexible pressure sensors with high sensitivity and good linearity are in high demand to meet the long-term and accurate detection requirements for pulse detection. In this study, we propose a composite membrane pressure sensor using polydimethylsiloxane (PDMS) and multiwalled carbon nanotubes (MWNTS) reinforced with isopropanol prepared by solution blending and a self-made 3D-printed mold. The device doped with isopropanol had a higher sensitivity and linearity owning to the construction of additional conductive paths. The optimal conditions for realizing a high-performance pressure sensor are a multiwalled carbon nanotube mass ratio of 7% and a composite membrane thickness of 490 μm. The membrane achieves a high linear sensitivity of −57.07 kΩ∙kPa^−1^ and a linear fitting correlation coefficient of 98.78% in the 0.13~5.2 kPa pressure range corresponding to pulse detection. Clearly, this device has great potential for application in pulse detection.

## 1. Introduction

In recent years, flexible pressure sensors have rapidly established their status as the key components for health monitoring, electronic skin, and wearable medical devices [1,2,3,4,5,6,7,8]. Pressure sensor refers to a device that can sense pressure transformation and output electrical signals. It can be simply divided into piezoelectric [9,10,11], piezoresistive [6,7,8,12,13,14,15], and capacitive [16,17,18,19]. Piezoelectric pressure sensors do not require an external power source, but they require complex metrological analysis and cannot perform static measurements. Capacitive pressure sensors can perform static and dynamic work, and their measurement results are highly repeatable, but it needs a complicated filtering system to reduce noise. Piezoresistive sensors do not require complex measurement analysis or complex data acquisition systems and are not sensitive to electromagnetic noise [20]. Therefore, the resistive pressure sensor has great advantages in health monitoring, especially the flexible piezoresistive pressure sensor obtained by adding a conductive filler to the elastic polymer matrix has attracted the attention of many researchers [21,22,23,24,25]. For example, this team used a flexible piezoresistive smart insole prepared by filling polydimethylsiloxane (PDMS) with multiwalled carbon nanotubes to monitor the plantar pressure of diabetic patients and analyze patient gait [21].

Pulse detection plays an important role in the field of health detection [26]. A large number of clinical studies have shown that pulse waves carry a lot of physiological information about cardiovascular diseases [27,28]. Different from the electrocardiogram, the pulse wave is caused by the arterial pressure wave, which can well-reflect the mechanical characteristics of the blood vessel wall [28]. The pulse signal is usually collected from the radial artery at the wrist. Traditional artificial pulse checking relies on the subjective experience of the doctor and cannot record the pulse wave in real time [29]. Currently, photoplethysmography (PPG) collects cardiovascular information based on changes in the light absorption of the blood vessel, which is the most widely used commercial technology for pulse measurement [30,31]. Nevertheless, it is easy for the PPG to collect inaccurate pulse signals due to the influence of ambient light variation and body movement [32].

Therefore, there has been much research using flexible pressure sensors for pulse detection. This team proposes a wearable device based on a carbon nanotube–polydimethylsiloxane [33]. The device has a linear correlation coefficient of 0.98 in the pressure range from 0.4 kPa to 14.0 kPa, which can be used for wrist pulse detection after preprocessing. Another team [34] proposes a pressure sensor made of PDMS with a lotus leaf surface structure and a graphene film. The linear sensitivity of the device is in the pressure range of 0~20 kPa and reaches 1.2 kPa^−1^. According to the information [2], the pressure range of pulse detection using the resistive pressure sensor is 0.2~3 kPa. Despite the promise of these complex structures, there still lacks a simple and low-cost method to prepare flexible piezoresistive pressure sensors with a high sensitivity and good linearity within the pulse detection pressure range.

**Figure 1:**
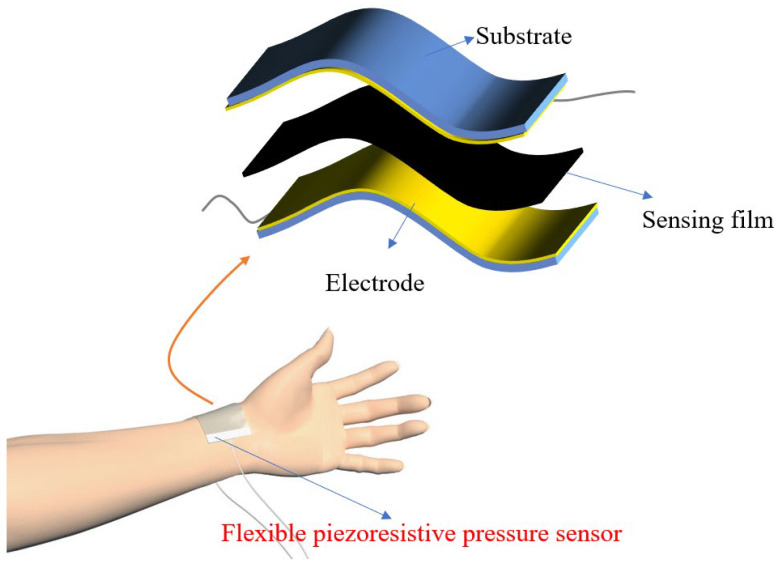
Schematic illustration of pulse detection based on pulse signal sensor.

In this paper, we prepare a flexible piezoresistive pressure sensor combined with a conductive composite membrane by doping multiwalled carbon nanotubes (MWNTS) into PDMS reinforced with isopropanol and self-cutting copper foil, as shown in Figure 1. The optimal conditions for preparing the pulse detection sensor are systematically explored from the organic solvent, electrode structure, filling concentration, and composite membrane thickness. In addition, a multipoint measurement system based on a flexible piezoresistive sensor array is proposed.

## 2. Materials and Methods

### 2.1. Materials

Multiwalled carbon nanotubes (MWNTS) with an outer diameter of 8~15 nm and a length of 50 µm were purchased from Chengdu Dingsheng Times Technology Co., Ltd., Chengdu, China. Analytical grade solvents isopropanol alcohol (IPA) and absolute ethanol were purchased from Chengdu Dingsheng Times Technology Co., Ltd., Chengdu, China. Two-part liquid silicone rubber (PDMS, code Sylgard 184) was provided by Dow Corning.

### 2.2. Preparation of PDMS/MWNTS Nanocomposites

**Figure 2:**
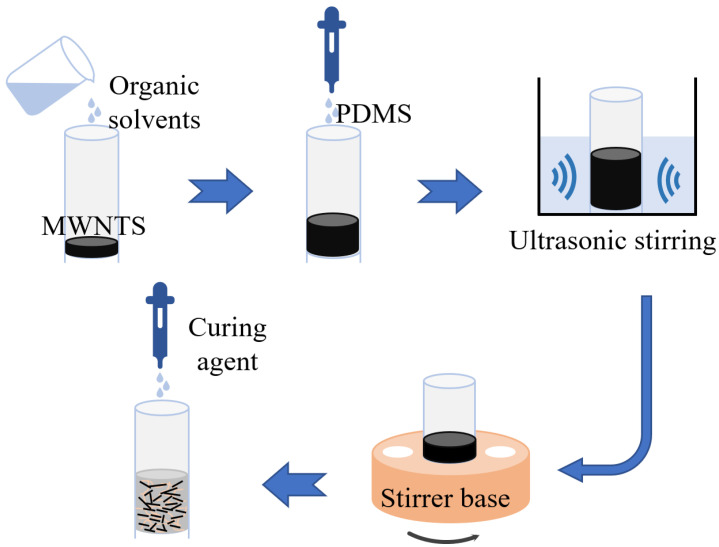
The preparation process of composite material mixed solution based on solution blending.

We spend more energy to completely disperse MWNTS into PDMS, considering the viscosity of PDMS. An organic solvent acts as a dispersing medium to predisperse the MWNTS by mixing and mechanical stirring, and later is mixed into the PDMS. Figure 2 shows the main steps of preparing the PDMS/MWNTS composite. Firstly, MWNTS were dispersed into isopropanol and mechanically stirred at 750 rpm for 5 min using a test tube shaker (Asone, Sinopharm Chemical Reagent Co., Ltd., Shanghai, China). The appropriate amount of PDMS was mixed to the dispersion via ultrasonic stirring with small ultrasonic cleaning machine (Asone, SB-120D, Sinopharm Chemical Reagent Co., Ltd., Shanghai, China) for 30 min at 42 kHz and mechanical stirring with test tube shaker for 40 min at 1000 rpm. The weight fraction ratio of MWNTS to PDMS is found to be optimized at 7%. Curing agent was added in the ratio 10:1 (dispersion: curing agent) and mechanically stirred for 5 min at 900 rpm to obtain the mixed suspension.

### 2.3. Fabrication of Piezoresistive Pressure Sensors

**Figure 3:**
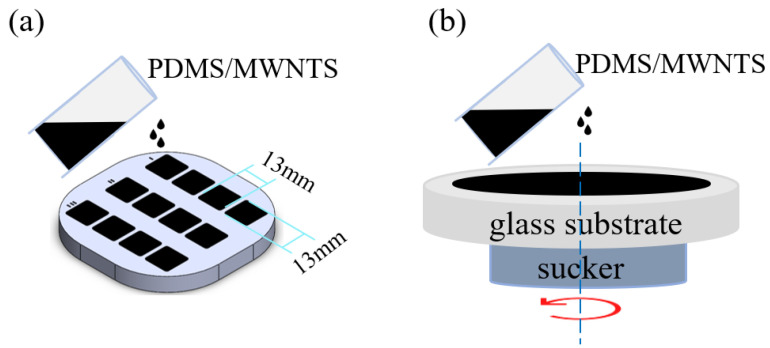
Schematic illustration of composite membrane prepared by (**a**) the 3D mold method and (**b**) the spin coating method.

Figure 3 shows the complete process of membrane formation. The 3D mold method and spin coating method were proposed to obtain the polymer composite membrane, respectively. For the 3D mold method, the 3D printed mold with 12 square slots for sensors of a side length of 13 mm and a depth of 500 µm was used to mold the sensors. The mixed suspension was transferred into a 3D-printed mold to cure for 30 min at 85 °C within a drying oven (DHG-9013A) in order to produce nanocomposite sheets (see Figure 3a). The composite membranes were removed from the mold after the curing process was completed. Figure 3b shows the spin coating method to obtain a thinner composite membrane because of the limitation of the 3D-printed mold. Firstly, the polymer mixture was dropped onto the glass substrate and placed in the homogenizer for different times at 1500 rpm to obtain the sensors with different thicknesses. After that, the glass substrate was taken out from homogenizer and placed on the heating plate for 20 min at 90 °C. The dimensions of the PDMS/MWNTS composite membranes prepared by both methods were cut to 10 × 10 mm. Finally, the prepared composite membrane was placed on a pair of self-cutting conductive copper foils to prepare a pressure sensor.

### 2.4. Characterization

**Figure 4:**
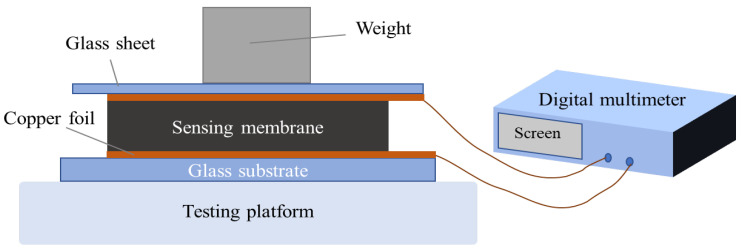
The schematic diagram of composite membrane characterization.

The performance of the sensors was characterized by measuring the change of the device resistance with using a digital multimeter (WEINSTEK GDM-9601) under the applied compressive force. Figure 4 shows a schematic diagram of the characterization of the composite membrane prepared in the experiment. The glass sheet placed on top of the sensing membrane was used to acquire accurate measurement results by evenly dispersing the external normal compressive force. Our study is to develop a piezoresistive sensor for pulse detection, so our focus is on testing the performance of the device under low pressure. The force applied to the sensor varied within the pressure range of 0.13~13.1 kPa by placing different weights. In the process of applying pressure, the resistance of the device was measured on two self-cutting conductive copper foil electrodes. The final change in the resistance of the device was the average value of the resistance changes of the sensor within 50 s. The standard deviation of each data point is also calculated to indicate the degree of dispersion of the resistance of the measured device. We systematically explored the optimal conditions for the preparation of pulse detection sensor in terms of four aspects: organic solvent, electrode structure, filling concentration, and composite membrane thickness. All data results were measured at room temperature conditions.

## 3. Results and Discussion

### 3.1. Sensor Design and Working Principle

**Figure 5:**
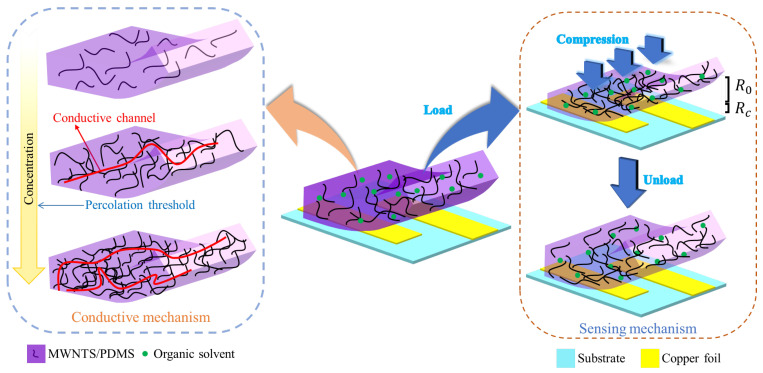
The conductive mechanism and sensing mechanism of pressure sensor.

Figure 5 presents the conductive mechanism and sensing mechanism of the flexible piezoresistive sensor. The sensor is made by assembling self-cutting conductive copper foil, MWNTS/PDMS composite membrane, and substrate. The conductivity of the composite material is related to the concentration of the conductive particles. The complete conductive channels will gradually form in the composite material as the filling concentration of the MWNTS increases, which results in a sharp increase in the conductivity of the composite material. The specific value of the filling concentration is called the percolation threshold. The higher the MWNTS filling concentration, the better the conductivity of the polymer due to the formation of more conductive channels. It is very important to select a suitable MWNTS fill concentration. At a high filler concentration, the composite membrane almost becomes a conductor, leading to insensitivity to external pressure. At a low filler concentration, there are very few conductive channels in the composite membrane, resulting in very high resistance. The number of new conductive channels is very limited under high pressure, resulting in an insensitivity to pressure. As a dispersion media, organic solvents can improve the pressure sensing performance of the PDMS/MWNTS composite membrane [35].

The resistance of the whole sensor is defined with the formula

(1) $$R_{s}=R_{c}+R_{0}$$

where $R_{c}$ is the contact resistance of the composite material membrane and the conductive copper foil and $R_{0}$ is the resistance of the composite material membrane. The resistance $R_{0}$ of the composite membrane decreases as one or more adjacent MWNTS tunnels are formed under the external pressure. When the compressive force is removed, the composite membrane returns to its original state and the sensor resistance also returns to its original value due to the high elasticity of the composite membrane. The sensitivity of sensor is expressed with formula

(2) $$S=\Delta R_{s}/\Delta P$$

where $\Delta R_{s}$ is the change value of the sensor resistance and ΔP is the change value of the applied compressive force.

### 3.2. The Characterization of Sensor Performance

During the characterization of the device, the impact of four different conditions on sensor performance is systematically explored to find the optimal conditions for preparing the pulse detection sensor. The four parameters evaluated are: (a) the influence of organic solvent on the sensor performance; (b) the influence of electrode structure on the sensor performance; (c) the influence of the MWNTS filler weight on the sensor performance; (d) the influence of the composite material membrane thickness on the sensor performance.

#### 3.2.1. The Impact of Organic Solvent

The working principle of the device we designed is based on the resistance mechanism. The resistivity of the MWNTS/PDMS composite material changes when a normal compressive force is applied to the sensing membrane. The electrical conductivity of the composite material is determined by the conductive paths formed by the carbon nanotubes in the PDMS matrix. The dispersibility of MWNTS in the polymer matrix is an important factor affecting the conductivity of composite materials. Therefore, whether the MWNTS can be completely uniformly dispersed in the PDMS matrix is a crucial issue. The use of organic solvents can promote the dispersion of the MWNTS well. In this work, we conducted pressure response tests on sensors prepared with isopropanol or ethanol absolute.

**Figure 6:**
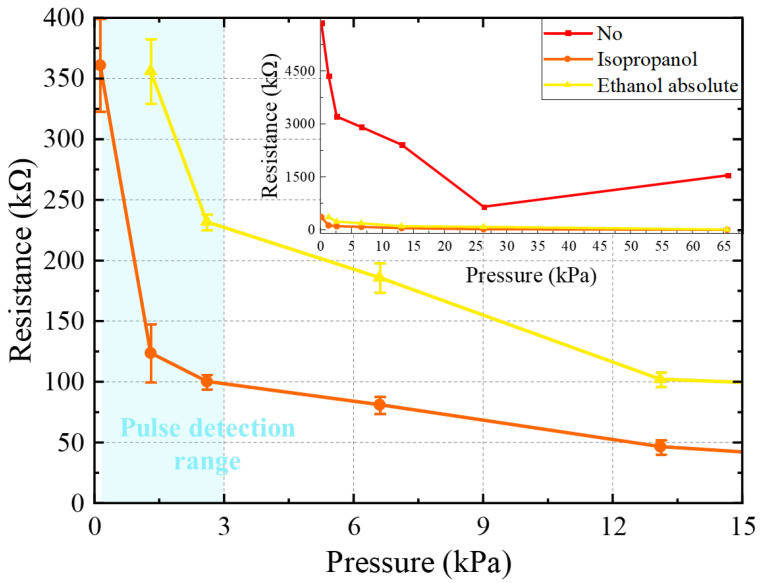
The pressure response of pressure sensors prepared with isopropanol, ethanol absolute, and nothing.

Figure 6 shows the pressure response results of the pressure sensor prepared with different organic solvents. The error bars for each data point represent the standard deviation of the five-times resistance of the measured device. The resistance of the pressure sensors prepared without organic solvents is an order of magnitude higher than that of the pressure sensors prepared with organic solvents at the same compression force (Figure 6 inner graph), indicating that the former have much fewer conductive paths than the latter. The number of conductive paths is determined by the MWNTS dispersion in the PDMS matrix, so the MWNTS dispersion in the pressure sensors prepared without the organic solvents is worse than that in the pressure sensors prepared with the organic solvents. The dispersion of the MWNTS inside the PDMS matrix is influenced by van der Waals forces, which cause the MWNTS to form nanotube bundles. The presence of organic solvents weakens the above effect, which is why the pressure sensors prepared with the organic solvents have lower resistance. In addition, the pressure response of the pressure sensors prepared without the organic solvents is unstable, further indicating that there is severe MWNTS agglomeration in this class of pressure sensors. The change in the conductive path in the composite membrane when the pressure sensor under the external load leads to a change in the pressure sensor resistance, and the poorly dispersed MWNTS pressure sensor leads to an unstable degree of conductive paths change, resulting in an unstable pressure response result. From Figure 6, we can see that the pressure response curve of the pressure sensor prepared with the isopropanol is similar to that of the pressure sensor prepared with the ethanol absolute, except that the former has a smaller resistance than the latter at the same pressure. This indicates that isopropanol has the greatest effect on promoting the dispersion of the MWNTS and forms more conductive paths in the PDMS matrix. Moreover, adding isopropanol to the device promotes a better pressure response performance in the pressure range corresponding to pulse detection (blue area in Figure 6). Therefore, we chose isopropanol as the dispersion medium for preparing composite membrane.

#### 3.2.2. The Impact of Electrode Structure

**Figure 7:**
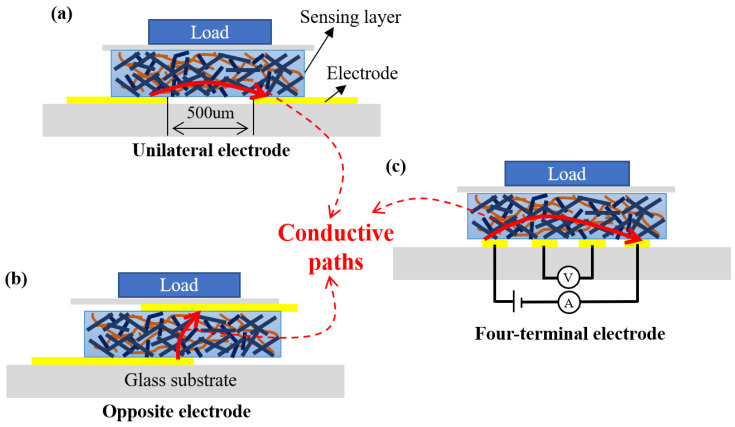
The schematic diagram of the electrode structure of (**a**) the unilateral electrode model, (**b**) the opposite electrode model, and (**c**) the four-terminal method.

The performance of the sensor is determined by the effective conductive path connected to the test circuit in the composite membrane, which is affected by the position and area of the electrode. In this section, the influence of the electrode position on the performance of the sensor is discussed. Figure 7 shows the schematic diagrams of the sensor composed of a different electrode structure on the unilateral, opposite, and four-terminal. Figure 7a shows the unilateral electrode model, where a self-cutting copper foil electrode with 500 μm is placed on one side of sensing layer. The opposite electrode model means that there are electrodes on both sides of sensing layer, and the electrode spacing is the thickness of the sensing layer as shown in Figure 7b. The four-terminal electrode method has four electrodes in total, which are placed on one side of sensing layer, as shown in Figure 7c. The two outermost electrodes form a power supply loop to provide current through the sensing layer. The inner two electrodes form a measurement loop to measure the voltage, and finally obtain the resistance of the composite membrane. Theoretically, this method can reduce the influence of the contact resistance of the composite membrane to obtain a more accurate composite membrane resistance.

**Figure 8:**
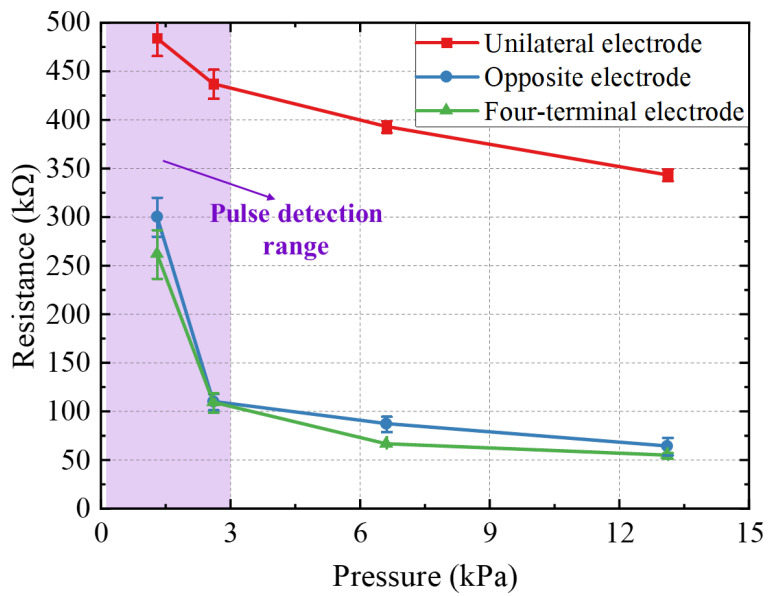
The results of pressure response of sensors with different electrode structure.

Figure 8 shows the pressure response results of sensors with the MWNTS filler concentration of 7% and a membrane thickness of 500 μm at different electrode positions. The error bars for each data point represent the standard deviation of the five-times resistance of the measured device. Under the same pressure, the resistance measured by the unilateral electrode model is 200~300 kΩ higher than the resistance obtained by the other two methods. The pressure response curves of the opposite electrode method and the four-terminal electrode method are similar. The opposite electrode model has more conductive paths connected into the test circuit than the unilateral electrode model, and the conductive circuit is changed more obviously under the applied normal pressure, resulting in greater sensitivity and lower resistance under low pressure. Compared with the unilateral electrode method, the four-terminal electrode method has a much lower resistance, which shows that it can reduce the influence of the contact resistance. In the four-terminal electrode method, in the high-pressure range, the change of the conductive path in the composite membrane reaches the limit and the pressure response becomes a smooth straight line. The resistance change of the single-side electrode model and the opposite electrode model is larger than that of the four-terminal electrode method. As the pressure increases, the contact resistance will still slowly decrease, and the resistance of the composite membrane will change to the limit. We choose the opposite electrode model as the electrode position of the sensor for pulse detection, considering the sensitivity and simplicity of the device and reducing the influence of contact resistance. The device has a very high sensitivity in the pressure range of pulse detection, as shown in the purple area in the figure.

#### 3.2.3. The Impact of MWNTS Filler Concentration

**Figure 9:**
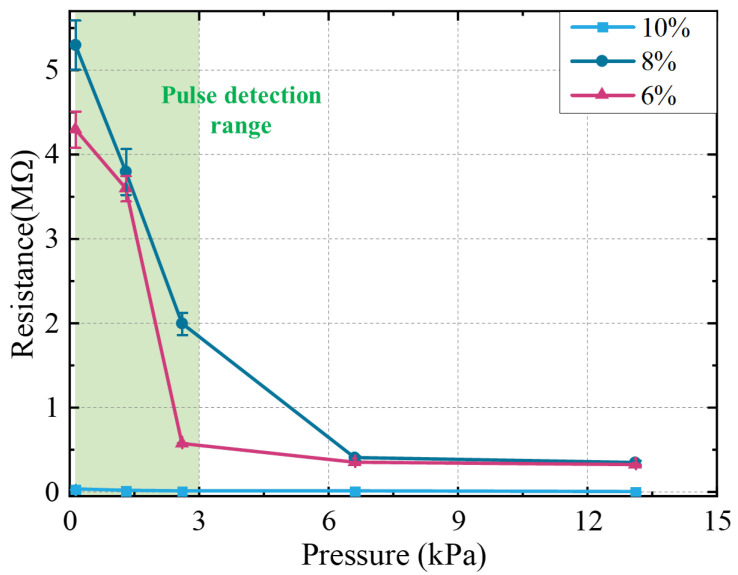
The pressure response of sensors with different MWNTS filler concentration.

The MWNTS/PDMS composite material exhibits resistance behavior, which is expressed as the resistance change of the composite material under the action of mechanical stimulation. The composite material conducts electricity because the MWNTS forms conductive paths in the PDMS matrix. Consequently, the composite material with a higher MWNTS filling concentration has an increased probability of forming conductive paths in the matrix, resulting in a higher conductivity and a lower resistance. The performance of the sensor was characterized by measuring the resistance of the devices with different filling concentrations of the MWNTS. Figure 9 shows the changes in the resistance of the devices consisting of 6, 8, and 10 wt% of the MWNTS under external pressure. The error bars for each data point represent the standard deviation of the five-times resistance of the measured device. The resistance of the sensor with 8% mass ratio of the MWNTS is smaller than that of the sensor with the 6% mass ratio of the MWNTS at the same external pressure, further indicating that higher filler concentrations form more conductive paths in the PDMS/MWNTS composite membrane. The resistance of the sensor with a 10% mass ratio of the MWNTS is essentially equal to zero, indicating that the PDMS/MWNTS composite membrane has become a conductor without pressure-sensitive properties at this filling concentration. The resistance changes of the device consisting of 6 wt% and 8 wt% of the MWNTS composite is more obvious than in the 10 wt%. The high mass ratio of carbon nanotubes forms a very large number of conductive paths in the device. The change of the conductive path is not obvious compared to the initial conductive path under compressive force. Therefore, its resistance change is much smaller than that of a device with a lower MWNTS concentration. In addition, the devices consisting of 6 wt% and 8 wt% MWNTS composite materials have very high sensitivity in the pressure range from 0.13 kPa to 3 kPa (green area in figure), and the sensitivity is very small in the high-pressure range. The devices prepared with a lower concentration of the MWNTS are beneficial to obtain a better stretchability. The devices consisting of 8 wt% MWNTS had better linearity in the low-pressure range than in the 6 wt%. Therefore, we chose the middle 7 wt% MWNTS mass ratio between 6 wt% and 8 wt% as the pressure sensor filler concentration for pulse detection.

#### 3.2.4. The Impact of Composite Membrane Thickness

**Figure 10:**
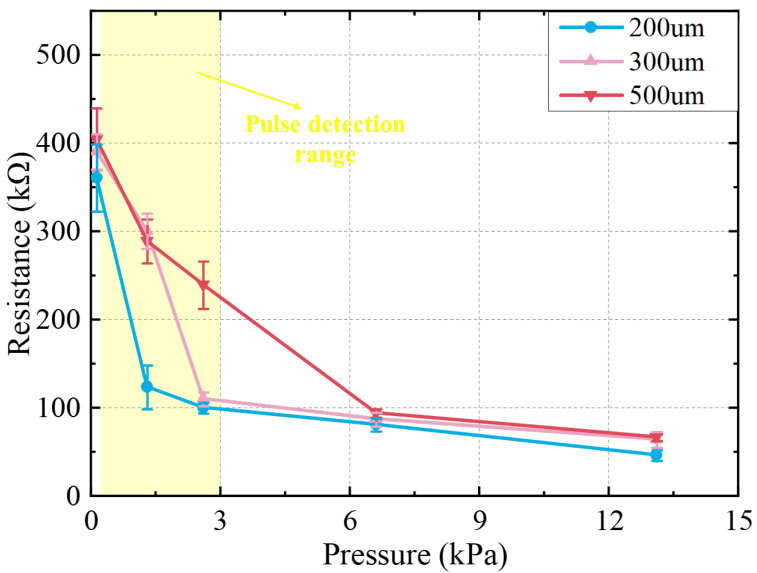
The pressure response of sensors with different composite membrane thicknesses.

Figure 10 shows the resistance changes of the sensors composed of the composite membrane with different thicknesses characterized under the same test conditions. The error bars for each data point represent the standard deviation of the five-times resistance of the measured device. The resistance of the device is small compared to a device with a larger composite membrane thickness because the smaller thickness of the composite membrane means the higher the probability of forming an effective conductive path between the electrodes. The resistance of the pressure sensors with three thicknesses changes rapidly in the pressure range of 0.13~6.6 kPa, and the resistance change is small in the range greater than 6.6 kPa. The smaller the thickness of the composite membrane, the higher the sensitivity in the low-pressure zone in the device (yellow area in Figure 10). When the external pressure is applied, the conductive paths of the device with a smaller thickness change more obviously, resulting in more resistance changes in the sensor. After the pressure is greater than a specific value, the degree of change of the conductive paths in the PDMS/MWNTS composite membrane tends to limit, leading to an insignificant change in the resistance with increasing pressure. We know that the resistance of the thinner membrane stabilizes earlier as the increase of external pressure from the picture because it is earlier to reach the limit of the conductive path change due to the smaller membrane thickness. As the 500 μm thick membrane has a more stable resistance variation in the pressure region of the pulse detection at the adjacent pressure test points, we chose a device with a composite membrane thickness of 500 μm for pulse detection.

#### 3.2.5. Quantitative Analysis for Optimal Conditions

**Figure 11:**
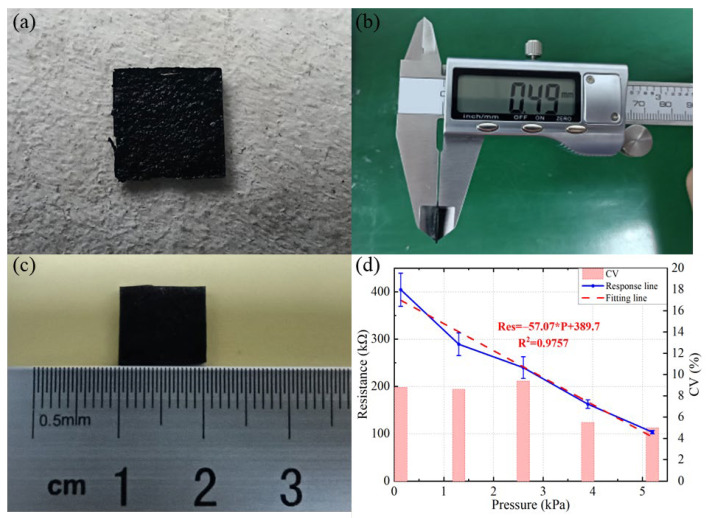
The optimal device in our work: (**a**) the real prototype of PDMS/MWNTS composite membrane; (**b**) the thickness measurement of the composite membrane; (**c**) the length measurement of the composite membrane; (**d**) the pressure response curve of the optimized flexible pressure sensor, where the dashed line is the linear fitting result, and the bar chart represents the coefficient of variation (CV) of the resistance measurement results at different pressures.

Finally, we prepared and quantitatively analyzed the optimized flexible pressure sensor. Figure 11a shows the real prototype of the PDMS/MWNTS composite membrane we prepared by solution blending. Figure 11b,c show the thickness and length measurements of the composite membrane, where the thickness of the optimized PDMS/MWNTS composite membrane is 0.49 mm and the side length is 10 mm. Figure 11d presents the pressure response curve of the optimized flexible pressure sensor from 0.13 kPa to 5.2 kPa. We show the standard deviation of each data point in Figure 11d, but this only characterizes the dispersion of the five resistance measurements at the same pressure and cannot be used to evaluate the dispersion of the resistance measurements at different pressures. We normalize the resistance measurements of the optimal device and use the coefficient of variation (CV) to characterize the dispersion of the resistance measurements of the optimal device at different pressures. The coefficient of variation is a dimensionless quantity defined as the standard deviation divided by the mean. From Figure 11d, it is known that the maximum coefficient of variation of the optimal device is 9.4% when the pressure is 2.6 kPa, and the coefficient of variation is less than this value for the rest of the pressures, further indicating that it is reasonable to use the average value instead of the resistance of the device at different pressures. The resistance value of our prepared composite membranes is 404.6 kΩ at 0.13 kPa, and the volume resistivity of the PDMS/MWNTS composite membrane is $8.3\times 10^{4}$ Ω∙m. In addition, we perform sensitivity calculations and linear fitting for the optimized sensor. Using the least square method to achieve the linear fitting of the curve, the calculated linear sensitivity is −57.07 kΩ∙kPa^−1^ and the linear correlation coefficient is 98.78% at 0.13 kPa~5.2 kPa. It can be drawn from the figure that our equipment with the isopropanol has a high sensitivity and a high linearity in the pressure range corresponding to the pulse detection.

### 3.3. The Prospect of Array Resistance Scanning Processing Circuit

**Figure 12:**
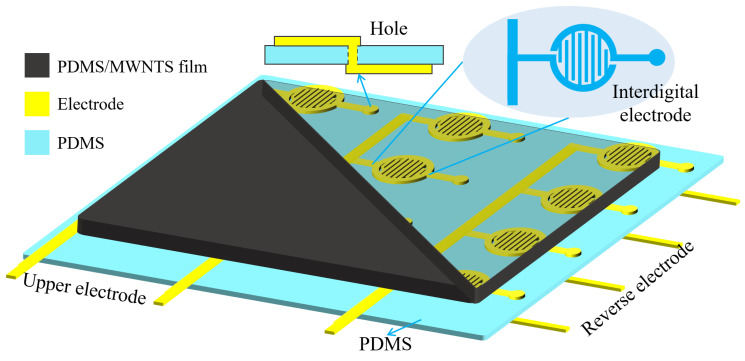
The schematic diagram of resistive pressure sensor array.

The flexible resistive pressure sensor array composed of the PDMS substrate, the array electrode, and the composite membrane will be prepared later, as shown in Figure 12. On the top side, each column of the circular interdigital electrodes is connected. Each electrode unit is designed with a circular interdigital structure. The through hole was inserted into the right side of each electrode to connect with the reverse electrode. The composite material membrane is placed on top of the upper electrode. On the reverse side, each row of electrodes is connected.

**Figure 13:**
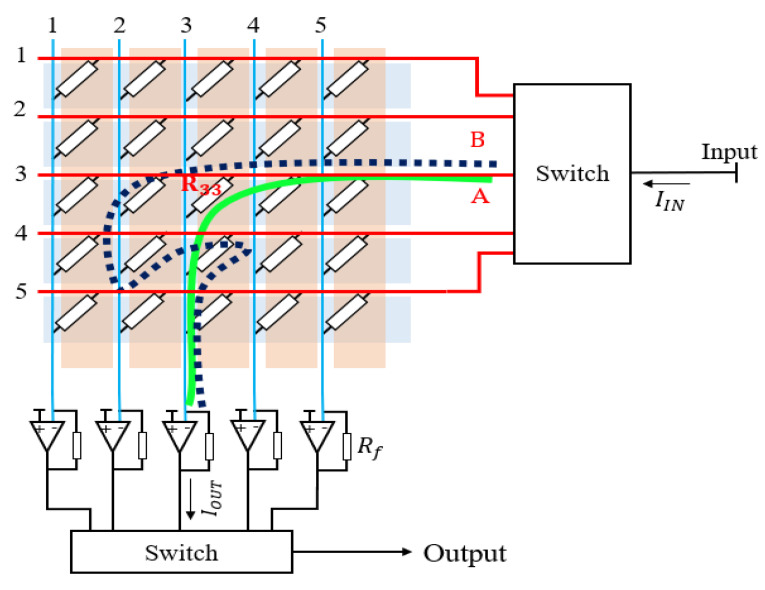
The schematic diagram of 5 × 5 resistance matrix scanning processing circuit. The blue rectangle and orange rectangle indicate five columns and five rows, respectively. A resistor exists between each row and column, and $R_{33}$ indicates the resistance between the third row and the third column. Letters A and B indicate the two conductive paths for measuring the resistance of $R_{33}$, respectively.

Figure 13 shows the working principle of the scanning processing circuit for the resistive sensor matrix. The crosstalk effect is the biggest influencing factor in the array resistance measurement [36,37,38]. For example, we want to measure the resistance $R_{33}$ on path A (green line) in the figure, but the crosstalk path B (dark blue line) is also connected to the measurement circuit, which affects the measurement of $R_{33}$. The zero-point compensation scanning processing circuit is built using the on–off of the switch and the virtual short of the amplifier to avoid the influence of the crosstalk path. Turning on the switches in the third row and the third column and turning off the switches in other rows and columns sets the voltage on the other rows and columns to zero when measuring $R_{33}$. By using the amplifier, not only can the nonmeasurement column voltage be zeroed, but also the signal can also be amplified. Multipoint measurement can be achieved by microcontroller control switches. When an external load is applied to the resistive pressure sensor array, the microcontroller controls the switch on and off for each row and column, the resistance values at each location on the sensor array are read out, and then calculates the value and location of the load.

## 4. Conclusions

In summary, we developed a low-cost resistive pressure sensor reinforced with isopropanol with high sensitivity and linearity in the pulse detection range based on a simple architecture. The device consists of self-cutting conductive copper foil and an MWNTS/PDMS composite material with isopropanol prepared by solution blending and a self-made 3D-printed mold as the top conductive composite material. Isopropanol could well-promote the dispersion of the carbon nanotubes in the composite membrane to obtain a flexible pressure sensor with a high performance and a good linearity. We studied the influence of the electrode structure, the MWNTS filling concentration, and the composite membrane thickness on the sensing performance of the device. Experimental data show that the flexible pressure sensor with isopropanol prepared under the optimal conditions has a very high sensitivity and linearity in the pulse detection range, indicating its tremendous potential applications in pulse detection. The optimized flexible resistive pressure sensor showed a high linear sensitivity of −57.07 kΩ∙kPa^−1^ and a high linear correlation coefficient of 98.78% at 0.13~5.2 kPa. These parameters can be further optimized to achieve higher sensitive devices. We believe that the sensors we report are very promising for pulse detection. Therefore, these sensors will be expanded into a matrix to achieve multipoint measurement and combined with artificial intelligence to achieve pulse signal analysis and processing in the future. By optimizing and integrating these sensors, they can be widely used in wearable devices, electronic skins, and medical prostheses.

## Data Availability

Not applicable.
